# Recanalization of a Petersen Defect After Prophylactic Closure: A Case of Internal Hernia Following Laparoscopic Distal Gastrectomy

**DOI:** 10.7759/cureus.86382

**Published:** 2025-06-19

**Authors:** Hiroki Makinodan, Kentoku Fujisawa, Tatsuya Fukui, Shusuke Haruta, Masaki Ueno

**Affiliations:** 1 Department of Gastroenterological Surgery, Toranomon Hospital, Tokyo, JPN

**Keywords:** internal abdominal hernia, laparoscopic surgery, petersen’s defect, petersen’s hernia, whirl sign

## Abstract

Petersen hernia (PH) is a type of internal hernia in which a portion of the small intestine protrudes through a defect located between the small bowel limbs, transverse mesocolon, and retroperitoneum after any type of gastrojejunostomy. The laparoscopic approach facilitates the occurrence of this type of hernia owing to the lack of postoperative adhesions, which otherwise help prevent bowel motility and herniation. Closure of this anatomical space, formed between the jejunal mesentery, transverse mesocolon, and retroperitoneum, has been shown to significantly lower the incidence of hernia and associated complications such as bowel obstruction and strangulation. We report the case of a 41-year-old woman who underwent laparoscopic distal gastrectomy with a Roux-en-Y reconstruction and prophylactic closure of the Petersen’s defect (PD) two years earlier for gastric cancer. She presented with abdominal pain and postprandial vomiting of three days' duration. Her abdomen was slightly distended, and tenderness was noted in the upper abdomen. Laboratory examination results were unremarkable. Contrast-enhanced CT revealed an internal hernia. Exploratory laparoscopy was performed, revealing a reopened Petersen space hernia of the common limb, with obstruction and dilatation of the biliary limb. The incarcerated bowel was repositioned, and there was no evidence of ischemia. The PD was closed using non-absorbable sutures. Awareness of this postoperative anatomical defect, including the possibility of its recurrence even when initially closed, is essential for appropriate management, given the nonspecific nature of its clinical and laboratory findings. Maintaining a low threshold for diagnosis and ensuring early surgical intervention are warranted to prevent serious complications related to bowel necrosis.

## Introduction

Petersen hernia (PH) is a clinically significant subtype of internal herniation in which small bowel loops protrude through the potential space bounded by the jejunal mesentery, transverse mesocolon, and retroperitoneum. This defect arises after gastrojejunostomy procedures, irrespective of the surgical indication (e.g., gastric cancer resection or Roux-en-Y (R-Y) gastric bypass) [1,2]. The laparoscopic approach, while offering advantages in postoperative recovery, paradoxically increases the risk of PH due to reduced adhesion formation, a phenomenon that normally limits bowel mobility and herniation in open surgery [3].

Contemporary evidence demonstrates that prophylactic closure of the Petersen space during index operations reduces the incidence of internal hernias. The rationale centers on obliterating the anatomical “mesenteric trap” that permits intestinal loops to migrate into the retroperitoneal compartment [1-6]. Left unaddressed, this defect predisposes patients to catastrophic complications such as bowel obstruction and strangulation with ischemia. Surgeons must maintain a high index of suspicion for this complication, particularly in patients presenting with nonspecific abdominal symptoms following R-Y reconstruction. Prompt imaging evaluation and early surgical intervention are critical to prevent severe outcomes such as bowel necrosis [7,8].

In this study, we encountered a case of Petersen defect (PD)-associated internal hernia despite prophylactic closure during gastric cancer surgery with R-Y reconstruction. This case highlights that internal hernias through the PD remain a clinical possibility, even when the defect has been surgically closed. Clinicians should incorporate this understanding into postoperative management strategies, recognizing that anatomical closure does not universally eliminate the risk of herniation.

## Case presentation

**Figure 1:**
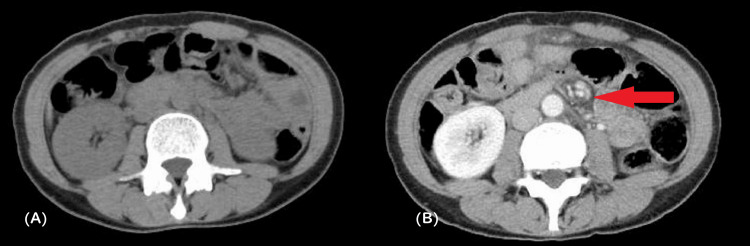
CT abdomen. 1A: Postoperative CT obtained 1 year after the initial gastrectomy showed no evidence of a whirl sign. 1B: Repeat CT at 1 year and 8 months postoperatively demonstrated a newly developed whirl sign (red arrow), indicative of small bowel rotation and mesenteric torsion.

A 41-year-old Japanese female with a history of laparoscopic distal gastrectomy (D2 lymph node dissection and antecolic R-Y reconstruction) performed 20 months prior for early gastric cancer (pT1aN0M0, Stage IA) presented to the emergency department with a three-day history of progressive epigastric pain. During the initial procedure, the mesenteric defects between the Y-limb and PD space were prophylactically closed using non-absorbable sutures. Surveillance endoscopy and imaging revealed no evidence of recurrence (Figure 1A).

The pain was described as acute-onset, colicky, and localized to the epigastrium (5/10 on the numerical rating scale), worsening postprandially and without alleviating factors. Associated symptoms included persistent nausea and non-bilious emesis. Notably absent were fever, hematemesis, melena, urinary symptoms, or changes in bowel habits. On admission, the patient’s height was 152 cm, and her body weight was 41.8 kg, representing a 21% decrease from her pre-gastrectomy weight (53.1 kg to 41.8 kg). Laboratory investigations revealed no significant abnormalities (Table 1). Abdominal CECT revealed a whirl sign, which was highly suggestive of an internal hernia (Figure 1B). Based on these findings, emergency surgical intervention was performed.

**Table 1:** Blood tests on admission. eGFR: Estimated glomerular filtration rate; CRP: C-reactive protein; Hb: hemoglobin; APTT: Activated partial thromboplastin time; ALP: Alkaline phosphatase; ALT: Alanine transaminase; GGT: Gamma-glutamyl transpeptidase; AST: Aspartate transaminase; LDH: Lactate dehydrogenase.

Test	Value	Normal range
Urea	7.0 mmol/L	8-20 mmol/L
Creatinine	0.62 mmol/L	0.46-0.79 mmol/L
Sodium	140 mmol/L	136-145 mmol/L
Potassium	3.9 mmol/L	3.6-4.8 mmol/L
eGFR	83.3 mL/min	>60 mL/min
CRP	0.01 mg/L	<0.15 mg/L
Hb	11.6 g/dL	11.6-14.8 g/dL
WBC	3.7 × 10³/μL	3.3-8.6 × 10³/μL
Platelets	327 × 10³/μL	158-348 × 10³/μL
Prothrombin time	27 sec	23.8-34.4 sec
aPTT	86.6 sec	70-130 sec
Amylase	89 U/L	44-132 U/L
Bilirubin	0.7 mg/dL	0.4-1.5 mg/dL
ALP	92 U/L	38-113 U/L
ALT	18 U/L	7-23 U/L
GGT	10 U/L	9-32 U/L
AST	20 U/L	13-30 U/L
Albumin	4.1 g/dL	4.1-5.1 g/dL
LDH	164 U/L	124-222 U/L

**Figure 2:**
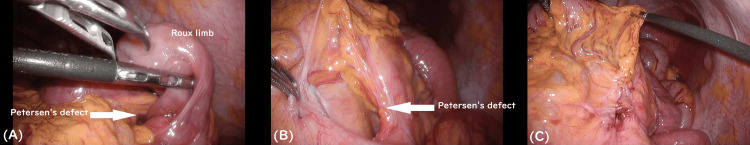
Intraoperative findings. A: The small intestine was found incarcerated in the Petersen’s defect (arrow), which had been closed during the previous surgery. B: Following reduction of the internal hernia, the previously incarcerated small intestine was repositioned into its normal anatomical location. C: The Petersen’s defect was subsequently re-closed using non-absorbable sutures.

The laparoscopic procedure was carried out using four abdominal ports. Intraoperative findings revealed the absence of adhesions or tumor recurrence within the abdominal cavity. Notably, the PD, which had been closed during the previous surgery, had reopened. During the operation, the non-absorbable barbed suture used for continuous closure of the Petersen defect in the initial surgery was found at the site of the incarcerated hernia, and dehiscence appeared to have occurred along the suture line. The afferent limb of the jejunum near the jejunojejunostomy site had herniated through this defect, resulting in a PH. The herniated small intestine was carefully reduced, and no evidence of ischemic changes was observed. The PD was subsequently re-closed using interrupted non-absorbable sutures (Figure 2). The total operative time was 44 minutes, and intraoperative blood loss was minimal. The postoperative course was uneventful. Oral intake was resumed on postoperative day 1, and the patient was discharged on postoperative day 6.

## Discussion

PH is a well-recognized but potentially life-threatening complication of laparoscopic distal gastrectomy with antecolic Roux-en-Y (R-Y) reconstruction. Although the R-Y technique is generally associated with favorable postoperative outcomes and a low incidence of complications, the PD remains a significant anatomical site that predisposes patients to internal herniation [3]. PH can still occur even when the PD is prophylactically closed during the initial surgery, as in the present case.

Several factors may contribute to the development of PH despite defect closure. Laparoscopic surgery results in fewer postoperative adhesions than open surgery [1]. Although this is advantageous in reducing adhesion-related complications, it may increase the risk of internal hernia by allowing greater mobility of the small intestine. In the present case, the absence of postoperative adhesions likely facilitated the herniation of the small bowel through the PD.

Second, significant postoperative weight loss is a recognized risk factor for internal hernia formation, particularly in patients undergoing bariatric R-Y gastric bypass, where the incidence of internal hernia ranges from 3 to 8%, with 15-60% of cases being attributable to the PD [9-11]. Rapid reduction in intra-abdominal and mesenteric fat can lead to the loosening or reopening of previously closed mesenteric defects. Although our patient was not obese, she experienced a 21% reduction in body weight postoperatively, which may have contributed to recanalization of the PD and subsequent hernia formation.

Third, technical factors such as incomplete closure of the PD during surgery cannot be excluded. Pan T et al. reported that the incidence of PH was significantly lower (0.3%) in cases where the PD was closed than in those without closure (1.3%); however, the risk was not entirely eliminated [6]. This highlights the importance of meticulous surgical technique and the need for standardized closure methods.

The choice of the reconstruction route is also relevant. The antecolic route is generally preferred over the retrocolic route because it is associated with a lower risk of internal hernia [11,12]. The antecolic approach creates two potential hernia sites: the PD and the mesenteric defect at the jejunojejunostomy site, whereas the retrocolic route introduces an additional defect in the transverse mesocolon. Therefore, prophylactic closure of all mesenteric defects is recommended to minimize the risk of hernia formation.

Diagnosis of PH remains challenging because of its nonspecific clinical presentation. Early symptoms often include postprandial abdominal pain and nausea; however, physical findings are frequently unremarkable. In this context, CECT is indispensable, with the “whirl sign” serving as a highly specific radiological indicator of internal hernia [13,14]. In our case, the diagnosis was facilitated by the identification of the whirl sign on CECT, despite the lack of definitive clinical findings.

In summary, this case highlights that PH can develop even after prophylactic PD closure, particularly in cases of significant postoperative weight loss and minimal adhesion formation following laparoscopic surgery. Surgeons should remain vigilant for this complication, especially in patients presenting with vague abdominal symptoms after R-Y reconstruction. Prompt imaging and early surgical intervention are essential to prevent serious outcomes such as bowel necrosis. Further studies are warranted to establish optimal closure techniques and to better understand the risk factors for PH in both bariatric and oncologic populations.

## Conclusions

This case demonstrates that PH can occur even after prophylactic closure of the PD during laparoscopic R-Y reconstruction. Surgeons should be aware that significant postoperative weight loss and reduced adhesion formation following laparoscopic surgery may increase the risk of internal hernia, even when the defect has been closed. Early diagnosis through imaging and prompt surgical intervention are essential to prevent serious complications such as bowel necrosis.
